# Impact of alcohol strength on attitudes and decisions concerning special occasion drinking during pregnancy

**DOI:** 10.1186/s12889-025-24234-6

**Published:** 2025-10-27

**Authors:** Sam Burton, Rebecca Monk, Emma Davies, Megan Goodier, Abigail K. Rose

**Affiliations:** 1 School of Psychology, Liverpool John Moores University, Liverpool, UK; 2 Department of Women’s and Children’s Health, School of Life Course and Population Sciences, Faculty of Life Sciences & Medicine, King’s College London, London, UK; 3 Department of Psychology, Edge Hill University, Ormskirk, UK; 4 Centre for Psychological Research, Oxford Brookes University, Oxford, UK

**Keywords:** Alcohol exposed pregnancy, Alcohol by volume, Risk perception, Alcohol harm

## Abstract

**Background:**

Special occasions are a risk factor for drinking during pregnancy. This study determined the impact of alcohol strength on attitudes around, and perceived harm of, drinking in pregnancy. If perceived harm decreases with lower strength alcohol, this may promote drinking when abstinence is recommended.

**Methods:**

Two online ‘special occasion’ vignette studies randomised female participants to one of three drink strength conditions (11%, 7.5%, 0% alcohol beverage volume [ABV]). In the study (*N* = 1128), participants were asked to imagine themselves or someone else choosing to consume the beverage when pregnant. Outcome measures assessed how harmful participants thought drink choice was, and the extent to which they agreed with the drink choice.

**Results:**

The standard and lower strength alcohol beverages were viewed as more harmful than the alcohol-free drink (*p <* .001), and participants agreed with the alcohol-free drink choice more than the standard and lower strength beverages (*p* < .001). Perceived harm was greater when rating own hypothetical alcohol use in comparison to rating observed hypothetical alcohol use (*p <* .01). Participants who reported drinking in their own pregnancy rated the alcohol choices as less harmful and more agreeable than participants who had not consumed alcohol in their own pregnancies (*p <* .001).

**Conclusions:**

Perceived harm, and the ability to apply the potential harms of drinking during pregnancy to one’s own circumstances, may be crucial in reducing the risk special occasions pose to alcohol exposed pregnancies. Public health campaigns should focus on facilitating this, compassionately explaining the risk of harms across a range of drinking behaviours, while explicitly tackling the stigma and shame women may experience around this public health issue.

## Introduction

Alcohol is the most widely used drug of women of typical child-bearing age, and prenatal alcohol use is the dominant preventable cause of birth defects and intellectual disabilities [1]. In 2016, the UK’s Chief Medical Officer guidelines were revised, adopting a ‘precautionary principle’ approach which recommended abstinence while trying to conceive and throughout pregnancy [2]. However, the UK continues to have one of the highest estimated rates of alcohol exposed pregnancies (AEP): 41.3%, compared to a pooled global estimate of 9.8% [3, 4]. Subsequently, the UK also has a high modelled prevalence rate of fetal alcohol spectrum disorder (FASD) (3.2%) [5], and it is estimated that 1 in every 13 pregnancies with some level of alcohol exposure results in FASD, with risk increasing with heavier drinking [6]. FASD is an umbrella term covering a range of neurodevelopmental issues and is associated with poorer life outcomes [1] and an estimated UK cost of £2 b p/a [7]. Low to moderate prenatal alcohol exposure is also associated with increased health risks to the child (e.g., mental health problems, low birth weight, preterm birth) [8–12]. FASD can also impair the wellbeing of birth mothers, who are often blamed and stigmatised for drinking during pregnancy [13]. There are common, inaccurate assumptions that birth mothers of children with FASD have alcohol use disorder, ‘wilfully’ drink during pregnancy despite known risks, and are unfit mothers [14]. This can result in poorer maternal mental health with increased experiences of depression, guilt, and stress [15, 16].

Given that 28.5% of UK women report drinking alcohol following pregnancy recognition [17] and the significant potential harms of AEP to the mother and child [18], it is important to identify factors that may increase likelihood of drinking following pregnancy recognition. Evidence from several countries identifies special occasions as one such risk factor [19–22]. Indeed, when Tsang and colleagues [23] surveyed pregnant women, they found that most women (61.3%) who drank after pregnancy recognition reported doing so during special occasions, and this included women who were non- and low-risk drinkers prior to pregnancy. Importantly, asking about special occasion drinking identified an additional 33.3% of respondents reporting alcohol use during pregnancy, who would not otherwise have been captured. This suggests that special occasions represent a risk to pregnant women that is likely under-reported and thus poorly understood.

Perception of harm is another important factor, with many health behaviour change interventions incorporating risk perception components [24]. Women often recognise that drinking in pregnancy can be harmful [21, 25, 26], whether or not they have been pregnant themselves [27]. Yet contradictory attitudes are common, and the strength or ‘potency’ of the drink appears to influence harm perception [21, 28]. This raises the possibility that increasing the availability of lower strength alcohol products, while intended as a harm reduction strategy [29], may have some unintended, negative consequences. There are several categories of ‘NoLo’ drink products, e.g., alcohol-free (≤ 0.05% ABV), de-alcoholised (≤ 0.5% ABV) and low alcohol (≤ 1.2% ABV). There are also a growing range of alcohol products with an ABV lower than ‘standard’ (e.g., beers under 4%, wines under 11% [if wine is under 8% it may be called a wine-based drink]) [30]. Importantly, there is a lack of awareness by the public in terms of what constitutes these categories [31], so it is possible that people may class lower than standard ABV beverages similarly to NoLo drinks and choose to drink them when they would normally abstain [32]. In the context of recommendations to avoid all alcohol consumption during pregnancy, the perceived risk of such beverages may be a factor in women’s decision making, raising concern as the market share of these products increases.

Supporting women to abstain from alcohol during pregnancy is a public health priority. Identifying factors that increase drinking during pregnancy or promote more accepting attitudes towards it can inform effective, evidence-based policies and public health interventions to reduce AEP. Social support can reduce and increase risky drinking in the general population [33, 34], and pregnant women often report drinking with friends or family [35]. Therefore, research should explore harm perception in both women who have been pregnant and those who have not, as both groups may influence pro/anti-attitudes of AEP and the latter could become pregnant in the future. However, there is an important distinction between these two populations, with those having experience of pregnancy likely to use their own alcohol use during this time to evaluate other pregnant people’s drinking behaviour. This is termed the ‘self-image bias’ [36], and has been demonstrated when assessing other’s drug use [37], therefore the impact of personal drinking habits needs to be considered. Additionally, when examining harm perceptions, it is important to consider the impact of ‘framing’. Evidence shows that individuals tend to underestimate the risks of their own alcohol use due to the ‘unrealistic optimism bias’, while judging other people’s drinking as more harmful [38, 39]. As such, it is useful to compare harm perception when judging personal versus other people’s alcohol use, to explore how best to frame public health messaging for maximal impact.

The current research used a vignette (scenario) method to determine, for the first time, attitudes and decision making around special occasion alcohol use during pregnancy, and the impact of alcohol strength on these outcomes. Based on the perceived reduced risk of harm from lower alcohol strength products, we hypothesised that individuals exposed to scenarios depicting lower strength alcohol consumption would judge drinking during pregnancy as less harmful and more acceptable than standard alcohol strength vignettes. By incorporating vignettes depicting the participant’s personal (hypothetical) alcohol use vs. that of another pregnant woman, we assessed the (unrealistic) optimism bias, and hypothesised that people would judge other pregnant woman’s alcohol use as most harmful. Finally, given the self-image bias, we hypothesised that within participants who had experience of pregnancy, their own alcohol use during pregnancy would reflect their harm and acceptability judgements of AEP in the vignettes.

## Methods

### Participants

All participants were aged 18 or over, were fluent in English, and self-identified as being assigned female at birth, in total 1149 participants were recruited. To ensure we were captured data from participants who were pregnant, as this may potentially affect attitudes, we purposively over-recruited women currently pregnant.

### Measures

#### Demographics

Age, ethnicity, sexuality, gender identity, relationship status, highest level of education, current occupation, average household income (before tax), history of pregnancy, number and age of children, UK area of residence. In study 3 participants were asked how many weeks pregnant they were.

#### Current alcohol consumption

The Timeline Followback (TLFB [40]), assessed weekly alcohol use. Using a diary format, participants were asked to record how many and what type of drink (e.g., large/small glass of wine, pint of beer) they had consumed over the past 14 days. Drinks were converted to units (1 UK unit = 8 g alcohol) and an average was calculated for weekly alcohol unit consumption.

#### Alcohol harm

The alcohol use disorders identification test (AUDIT [41]) assessed alcohol use and potentially harmful drinking behaviour (10 items). Scores indicate 0–7: low risk drinking, 8–15: increasing risk, 16–19: higher risk, 20 + possible dependence. For women it is recommended that low risk drinking is scored 0–6.

#### Alcohol use and pregnancy

Participants who indicated they were currently or historically pregnant were asked whether they changed their drinking habits (increased, no change, decreased, abstained) during different stages (3 months before pregnancy, 0–2 weeks, 3–6 weeks, 7–12 weeks, 12–26 week [second trimester], from week 27 [third trimester]). Participants were also asked in what contexts they had consumed alcohol during pregnancy. Several fixed options were given (e.g., special occasions [wedding, party], special periods [Christmas, Easter], with friends when out, with partner at home, when alone etc.) as well as free text options.

#### Vignette/Scenario

A short scenario described a woman called Sarah attending her friend’s wedding reception or as if the participant was attending as themselves, in both they are pregnant. Participants were randomly assigned to a vignette. As the vignette progressed, accompanying images were included to help the participant imagine the scenario (e.g., wedding marquee, people celebrating). The atmosphere was described as exciting and fun, with the individual enjoying spending time with friends. The scenario explains that the group decides to go to the bar to get drinks and everyone chooses to get a glass of sparkling wine to celebrate. The vignette states that the individual wants to join her friends and then introduces the information that the individual is pregnant. The vignette states that the individual asks the bar person if there is an alcohol-free sparkling wine available. At this point, participants are randomised to one of three drink availability conditions: standard 11% ABV, lower strength 7.5% ABV, and alcohol free 0.0% ABV. In each condition, Sarah decides to accept the drink that is available.

#### Attitudes around choice (primary outcome)

Following the end of the vignette, participants were asked two questions, ‘Do you agree with Sarah’s/your drink choice?’ and ‘To what extent do you think Sarah’s/your choice may harm her/your baby?’. Participants responded on a sliding scale from 0 (not at all) to 100 (very much).

### Procedure

The study recruited participants through social media sites (e.g., Twitter, Facebook), those taking part on social media were not offered incentives due to risk of fraudulent participants [42, 43], and Prolific (an online research recruitment platform). Interested individuals clicked on a link, which took them to the Participant Information Sheet. After providing online consent, participants provided demographic information, before reading the vignette and completing all questions in the order provided above (i.e. personal drinking habits were recorded after the vignette task to avoid these scales influencing responses). Sections on personal drinking habits included a statement that we made no judgement on participant’s alcohol choices. Two attention checks were distributed throughout the study. A debrief at the end provided guidelines on alcohol use during pregnancy and signposting to further information.

### Analysis

Participants were removed who did not pass attention checks (*n* = 21), 33 had missing data and was removed, leaving a final combined sample of 1095. Analysis was performed in R studio using the dplyr packages. Between subjects ANOVA’s were applied using the aov function in R. Independent variables were scenario (2 levels: self and other person), alcohol (three levels: no alcohol, low alcohol, standard alcohol) and drank in pregnancy (two levels: consumed alcohol, consumed no alcohol). Dependent variables were perceived harm of consuming alcohol (scored 0-100), and extent of which the participant agrees with the drink choice (scored 0-100). Sub-group analysis was conducted on those who were currently pregnant, as a sensitivity analysis.

### Ethics statement

Participants provided online informed consent: a tick box stating they had read/understood the participant information sheet, met inclusion criteria, and agreed to take part in the study. Only after this consent was provided did the study launch (via Qualtrics). The studies received ethical approval from the Psychology Research Ethics Committee at Liverpool John Moores University. Data collection occurred between 23/01/2022-21/12/2024.

## Results

### Participant characteristics

There were no significant differences between observed and self-rated vignette demographics (see Table 1). There were also no differences in demographics (*p* >.05); mean age (standard deviation) was 39.40 (± 12.91) years, and most participants were white (76.21%), married/cohabiting (58.13%), heterosexual (81.19%), and employed (64.68%) (see Table 1 for full demographic breakdown). Of the respondents who reported having been pregnant, 19.42% reported alcohol use at some point in pregnancy.

**Table 1:** Descriptive characteristics of the sample, split by study and total, displaying means (standard deviations), median (interquartile range) and n (%)

	Self-Vignette	Observed Vignette	Total
	(*n* = 581)	(*n* = 514)	(*n* = 1095)
Age			
Mean (SD)	37.62 (12.80)	36.98 (10.89)	37.31 (11.94)
Median (IQR)	34.00 (17.00)	34.00 (11.00)	34.00 (14.00)
Weekly alcohol units			
Mean (SD)	8.01 (15.48)	5.83 (11.21)	6.99 (13.70)
Median (IQR)	2.07 (9.71)	0.71 (9.71)	1.34 (8.52)
AUDIT			
Mean (SD)	4.99 (5.63)	4.55 (4.53)	4.78 (5.15)
Median (IQR)	3.00 (6.00)	3.50 (6.00)	3.00 (6.00)
n harmful drinkers (%)	83.00 (14.29%)	121.00 (23.54%)	204.00 (18.63%)
n hazardous drinkers (%)	15.00 (2.58%)	4.00 (0.78%)	19.00 (1.74%)
Ethnicity			
Any Other	4 (0.69%)	4 (0.78%)	8 (0.73%)
Asian - British	25 (4.30%)	19 (3.70%)	44 (4.02%)
Asian - Other	11 (1.89%)	5 (0.97%)	16 (1.46%)
Black - British	29 (4.99%)	8 (1.56%)	37 (3.38%)
Black - Other	39 (6.71%)	4 (0.78%)	43 (3.93%)
Mixed - Any	14 (2.41%)	15 (2.92%)	29 (2.65%)
White - Other	50 (8.61%)	46 (8.95%)	96 (8.77%)
White British	401 (69.02%)	404 (78.60%)	806 (73.61%)
Missing	29 (4.99%)	13 (2.53%)	43 (3.93%)
I prefer not to answer this question	4 (0.69%)	4 (0.78%)	8 (0.73%)
Relationship Status			
Divorced or separated	28 (4.82%)	17 (3.31%)	45 (4.11%)
I prefer not to answer this question	2 (0.34%)	2 (0.39%)	4 (0.37%)
In a relationship (not co-habitating)	74 (12.74%)	46 (8.95%)	121 (11.05%)
Married or co-habitating	367 (63.17%)	360 (70.04%)	727 (66.39%)
Single	97 (16.70%)	75 (14.59%)	172 (15.71%)
Widowed	4 (0.69%)	1 (0.19%)	5 (0.46%)
Missing	34 (5.85%)	21 (4.09%)	56 (5.11%)
Education			
Doctorate degree	40 (6.88%)	19 (3.70%)	59 (5.39%)
Master’s degree	128 (22.03%)	99 (19.26%)	228 (20.82%)
Bachelor’s degree	210 (36.14%)	200 (38.91%)	410 (37.44%)
Professional degree	16 (2.75%)	10 (1.95%)	26 (2.37%)
Trade/technical/vocational training	40 (6.88%)	63 (12.26%)	103 (9.41%)
Secondary School/College (e.g. A’Level)	97 (16.70%)	77 (14.98%)	174 (15.89%)
Secondary school (e.g. GCSE)	42 (7.23%)	39 (7.59%)	81 (7.40%)
Primary school	2 (0.34%)	0	2 (0.18%)
Other, please specify	4 (0.69%)	4 (0.78%)	8 (0.73%)
Prefer not to answer	1 (0.17%)	0	2 (0.18%)
Missing	26 (4.48%)	11 (2.14%)	37 (3.38%)
Employment			
Employed	395 (67.99%)	353 (68.68%)	749 (68.40%)
Self-employed	44 (7.57%)	36 (7.00%)	80 (7.31%)
Maternity leave	30 (5.16%)	24 (4.67%)	54 (4.93%)
Stay at home mum/homemaker	39 (6.71%)	50 (9.73%)	89 (8.13%)
Retired	27 (4.65%)	18 (3.50%)	45 (4.11%)
Unable to work	13 (2.24%)	9 (1.75%)	22 (2.01%)
Unemployed	21 (3.61%)	20 (3.89%)	41 (3.74%)
Prefer not to answer	11 (1.89%)	5 (0.97%)	16 (1.46%)
Missing	26 (4.48%)	7 (1.36%)	34 (3.11%)
Country of Residence			
United Kingdom	576 (99.14%)	513 (99.81%)	1090 (99.54%)
Other	8 (1.38%)	3 (0.58%)	12 (1.10%)
Missing	22.00 (3.79%)	6 (1.17%)	28 (2.56%)
Sexuality			
Heterosexual	497 (85.54%)	437 (85.02%)	935 (85.39%)
Bisexual	33 (5.68%)	46 (8.95%)	79 (7.21%)
Lesbian/Gay	18 (3.10%)	13 (2.53%)	31 (2.83%)
Other	9 (1.55%)	6 (1.17%)	15 (1.37%)
Prefer not to say	14 (2.41%)	11 (2.14%)	25 (2.28%)
Missing	35 (6.02%)	9 (1.75%)	45 (4.11%)
Gender			
Woman	574 (98.80%)	509 (99.03%)	1084 (99.00%)
Transman	4 (0.69%)	0	4 (0.37%)
Gender fluid	0	1 (0.19%)	1 (0.09%)
Non-binary	0	3 (0.58%)	3 (0.27%)
Other	1 (0.17%)	2 (0.39%)	3 (0.27%)
Prefer not to say	1 (0.17%)	0	1 (0.09%)
Missing	26 (4.48%)	7 (1.36%)	34 (3.11%)

Descriptive characteristics of the sample, split by study and total, displaying means (standard deviations), median (interquartile range) and n (%)

### Perceived harm analysis

**Fig. 1:**
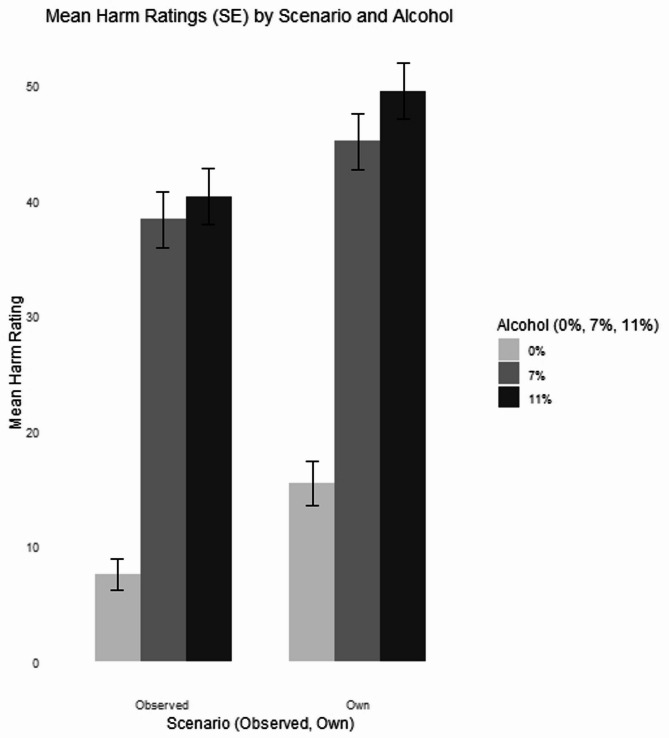
Alcoholic (7.5 and 11%) drinks were perceived as more harmful than alcohol-free drinks. Perceived harm scores were higher in the ‘own’ drinking scenario compared with the ‘observed’ drinking scenario

Between subjects ANOVA was applied with independent variables of scenario (two levels: own and observed drinking scenarios), alcohol (three levels: no alcohol, lower alcohol, and standard alcohol) and drank in pregnancy (two levels: consumed alcohol, consumed no alcohol), and the dependent variable of perceived harm of the vignette’s drink choice on the unborn baby (see Fig. 1). There was a significant main effect of scenario on perceived harm, F(1,693) = 22.35, *p* <.001, h^2^_p_ = 0.03. Contrary to our hypothesis based on the unrealistic optimism bias, those in the ‘own drinking’ scenario (M = 36.74, SD = 35.01) reported higher levels of perceived harm than those in the ‘observed drinking’ scenario (M = 29.04, SD = 31.85, *p <*.001). Additionally, alcohol was a significant main effect, F(2, 693) = 46.07, *p <*.001, h^2^_p_ = 0.12. Against our hypothesis, both standard (M = 45.17, SD = 33.16) and lower strength alcohol (M = 41.91, SD = 33.05) were viewed as significantly more harmful to the unborn baby than non-alcohol drinks (M = 11.78, SD = 23.48, *p <*.001), but there was no significant difference between lower or standard strength alcohol (*p* =.373).

Of participants who had experienced pregnancy but were not currently pregnant (*n* = 409), there was a main effect of drinking in pregnancy, F(1,693) = 16.92, *p* <.001, h^2^_p_ = 0.02. In line with our hypothesis based on the self-image bias, those who consumed alcohol during pregnancy (M = 20.12, SD = 27.18) viewed the consumption of alcohol as significantly less harmful to the unborn baby than those who had not consumed alcohol during pregnancy (M = 32.96, SD = 34.78, *p* <.001).

Sub-group analysis was carried out on those who were currently pregnant (*n* = 296). Between subjects ANOVA was applied with independent variables of scenario (two levels: own and observed drinking scenarios) and alcohol (three levels: no alcohol, lower alcohol, and standard alcohol), and the dependent variable of perceived harm of the vignette’s drink choice on the unborn baby. There was a significant main effect of alcohol, F(2,294) = 23.31, *p* <.001, h^2^_p_ = 0.14, on harm perception. Standard (M = 46.70, SD = 33.51) and lower strength alcohol (M = 43.35, SD = 32.36) were viewed as significantly more harmful to the unborn baby than non-alcohol drinks (M = 19.37, SD = 31.98, *p <*.001), but there was no significant difference between lower or standard strength alcohol (*p* =.774). There was a significant interaction between scenario and alcohol, F(2,294) = 4.07, *p* <.05, h^2^_p_ = 0.03. Post hoc analysis showed a significant difference between perception of harm for no alcohol wine (0% ABV) based upon observing (M = 7.92, SD = 21.32) and own (M = 29.33, SD = 36.31), *p* <.001, all other comparisons were non-significant (*p’s* > 0.996).

### Agreement with choice analysis

**Fig. 2:**
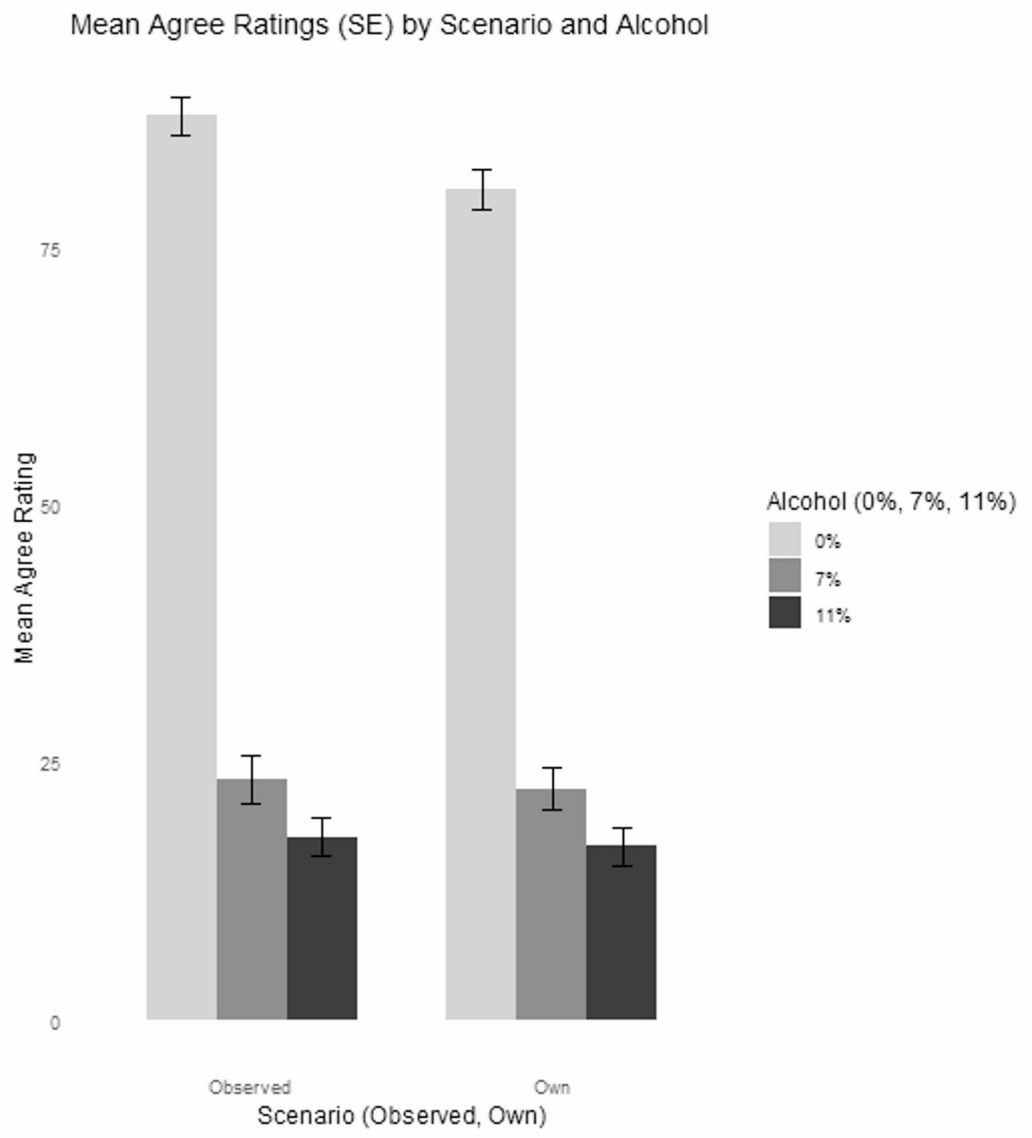
Participants agreed more with the alcohol-free choice compared with either the lower (7.5% ABV) or standard (11% ABV) alcoholic drink choices

Between subjects ANOVA was applied with independent variables of scenario (two levels: own and observed drinking scenarios), alcohol (three levels: no alcohol, lower alcohol, and standard alcohol) and drinking in pregnancy (two levels: consumed alcohol, consumed no alcohol), on the dependent variable of extent of which participants agreed with the drink choice (see Fig. 2). There was a main effect of scenario, F(1,694) = 11.65, *p* <.001, h^2^_p_ = 0.02, those who observed drinking (M = 42.49, SD = 40.99) agreed with alcohol use significantly more than those who rated their own drinking (M = 39.93, SD = 39.59, *p* <.001). There was a significant main effect of alcohol, F(2,694) = 293.84, *p* <.001, h^2^_p_ = 0.46. No alcohol drinks (M = 84.02, SD = 25.82) were significantly more agreeable than lower alcohol (M = 22.85, SD = 28.95, *p* <.001) and standard alcohol drinks (M = 17.23, SD = 24.87, *p* <.001), but there was no significant difference between lower and standard strength alcohol (*p* =.181).

There was a main effect of drinking in pregnancy, F(1,694) = 65.79, *p* <.001, h^2^_p_ = 0.09, those who consumed alcohol during pregnancy (M = 55.53, SD = 36.09) reported higher levels of agreement with the drink choice than those who had not consumed alcohol during pregnancy (M = 36.65, SD = 41.16, *p* <.001).

Sub-group analysis was carried out on those who were currently pregnant (*n* = 296). Between subjects ANOVA was applied with independent variables of scenario (two levels: own and observed drinking scenarios) and alcohol (three levels: no alcohol, lower alcohol, and standard alcohol), and the dependent variable of perceived harm of the vignette’s drink choice on the unborn baby. There was a significant main effect of alcohol, F(2,294) = 147.06, *p* <.001, h^2^_p_ = 0.50, on agreement perception. Standard (M = 16.56, SD = 22.74) and lower strength alcohol (M = 21.29, SD = 29.73) were viewed as significantly less agreeable to consume than non-alcohol drinks (M = 77.25, SD = 31.89, *p <*.001), but there was no significant difference between lower or standard strength alcohol (*p* =.454). There was a significant interaction between scenario and alcohol, F(2,294) = 3.47, *p* <.05, h^2^_p_ = 0.02. Post hoc analysis showed a significant difference between perception of agreeableness for no alcohol wine (0% ABV) based upon observing (M = 86.00, SD = 26.36) and own (M = 69.63, SD = 34.48), *p* <.05, all other comparisons were non-significant (*p’s* > 0.996).

## Discussion

Supporting women to abstain during pregnancy is a public health priority which can substantially benefit both mother and child wellbeing and health [44, 45]. Population-wide strategies have identified lower strength alcohol products as one way to reduce alcohol harms [29]. However, it is possible that if lower strength alcohol is perceived as less harmful, then some individuals may be more likely to drink alcohol when, typically, they would choose to abstain (e.g., in pregnancy). It is also known that special occasions can be a time when drinking in pregnancy is more likely [23]. This study used a novel vignette paradigm to explore perceived harm of drinking alcohol at varying strengths during pregnancy, and the extent to which women agreed with drinking choices during a special occasion.

Indicating that people draw distinctions between products when considering what to consume during pregnancy, we found (as expected) that both standard (11% ABV) and lower (7.5% ABV) alcohol products were perceived as more harmful than the alcohol-free beverage, and people agreed less with the drink choice when it contained any alcohol. Unexpectedly, there was no difference in perceived harm or agreement between the standard and lower ABV drinks. The precautionary principle of abstinence was introduced in the UK in 2016 and, as part of standard care, midwives should be providing this information at antenatal appointments. As such, our findings may be viewed as promising evidence that the recommendations to abstain entirely throughout pregnancy maybe influencing women, and that lower alcohol products are being placed in the same category of harm as products of standard ABV, even in the context of a special occasion (which is a known risk factor for drinking in pregnancy [23]). On the other hand, certain groups of women, e.g. those with alcohol use disorder, can find abstinence very difficult to achieve [35]. Although controversial and beyond this study, future research may look at the potential for harm reduction strategies in complex needs group, which incorporate low alcohol products, perhaps as part of a stepped treatment approach towards the recommended abstinence goals.

Also affording a somewhat optimistic view, while there were no differences in agreement with drink choice, participants who were asked to imagine their own consumption in the scenario reported higher levels of perceived harm than the participants asked to assess another person’s drinking. In a manifestation of the (unrealistic) optimism bias [38, 39], it has been shown that people tend to underestimate risk of harms from their own alcohol use, while judging other people’s drinking as more harmful [39, 46, 47], yet our current finding suggests this is not the case when judging pregnancy-related alcohol harm. This fits with a study that found a lack of unrealistic optimism bias in South African pregnant women regarding risks of drinking in pregnancy [48]. Indeed, in the context of pregnancy, self-perceptions of harm may be magnified in contrast to perceived harm to others, perhaps because fetus protection is a primary motive for not drinking during pregnancy [25, 49–51]. Our findings therefore suggest that asking women to evaluate potential alcohol risks in a more personal way (i.e., harm to your own pregnancy) may be an effective strategy to magnify perceived potential harms and thus reduce AEP.

Of more concern, although expected, is our finding that respondents who had consumed alcohol during their own pregnancies tended to perceive the consumption of alcohol in the vignettes as less harmful and more agreeable than respondents who did not drink during their own pregnancy. According to the self-image bias, we judge others by our own yardstick [36] and this has been found to apply to assessment of other people’s drug use [37]. Evidence suggests that some pregnant women and mothers are not convinced that low level alcohol use during pregnancy is harmful, they find the evidence and information given confusing, inconsistent and/or incorrect, and some believe that abstinence messaging is patriarchal [25, 26, 49]. Women, who had previously been pregnant but weren’t currently, judged drinking in pregnancy to be less harmful, they may be more likely to drink when pregnant but may also project that belief onto others in a similar situation. This is in contrast to women who were currently pregnant who viewed all alcohol consumption as harmful in comparison to no alcohol alternatives. This could have implications for pregnant women who may be subjected to social pressures during special occasions where normative affordances for consumption are particularly high [52, 53].

There are limitations to this study. First, we specified ABV % based on products that are currently on the market to make the results more applicable, however, it is not known whether there is a ‘tipping point’ at which ABV % is perceived as significantly more or less harmful. If such a tipping point exists, this may differ across beverage type. For instance, we used wine in the current study, but some women perceive risk from wine consumption as lower than other types of alcohol when pregnant [21, 52, 54, 55], and use type of alcohol rather than strength to evaluate risk [21]. Future research should use different types of alcohol (e.g., beer) in combination with varying ABVs, in order to further explore the interplay between beverage type and strength when it comes to drink selection (and risk perception) during pregnancy. Second, we focused on a single drinking occasion during a celebratory event, because special occasion drinking is a known risk for drinking in pregnancy. However, evidence shows that many women feel that low level, occasional alcohol use is acceptable during pregnancy [21, 25]. Given the evidence that risk of fetal alcohol harms is greater as levels of consumption increase and is particularly associated with binge drinking [56, 57], these attitudes are understandable. It is therefore possible that perceived harm and agreement would differ if we had included more ‘every day’ drinking scenarios. Third, we recruited women with and without experience of pregnancy. Although non-pregnant women are not the immediate target audience of this research, their inclusion is important. Better understanding of the factors that influence women’s attitudes and decisions around alcohol use behaviour during pregnancy can inform prevention strategies, either for women who become pregnant in the future and/or which incorporate components of social support/transmission of health information and advice. Last, we recruited a convenience sample which reported low levels of alcohol use, and we cannot assume heavier drinkers would respond in a similar way.

Future research can overcome these limitations by assessing perceptions towards a wider variety of alcohol strengths and products (e.g., wine/beer/spirits), across different situations, and by comparing different subpopulations of drinkers to develop a more nuanced understanding of this issue. This is important given the finding that people who consume higher levels of alcohol use may underestimate how harmful alcohol can be [58]. Future research could also use ecological momentary assessment tools to assess how drinking attitudes and harm perception may differ across pregnancy.

We would highlight, that despite our participant group’s low level of current drinking, 19.42% reported some level of drinking in pregnancy, aligning with recent estimates [59]. This finding confirms that alcohol exposed pregnancy in the UK is a significant public health issue and not something restricted to pregnant people with pre-existing hazardous or harmful drinking behaviours. We would also argue that any strategies to increase prevalence rates of abstinence during pregnancy carefully consider women’s perceptions of harm across drinking levels, and work with women to develop ways to justify the precautionary principle which focuses on supporting the health and wellbeing of the woman, as well as the child.

This novel study suggests that lower strength alcohol products are still perceived as harmful when considering special occasion drinking during pregnancy. This means that lower strength alcohol products may not be a risk for alcohol use in pregnancy, although we have outlined recommendations for future research to confirm this. This work also reinforces the importance that public health campaigns to reduce AEP should be framed so that women can understand that potential harms are directly applicable to their own drinking behaviours and pregnancy, and aligns with efforts to ensure women do not feel judged or stigmatised for their behaviour, and that compassionate framing supports women’s wellbeing.

## Data Availability

Data can be accessed on the following link on the OSF repository https://osf.io/kpq63/?view_only=681f9a50c4f4429ea3219b9dc521e875.
